# Expression of concern: subfunctionalization reduces the fitness cost of gene duplication in humans by buffering dosage imbalances

**DOI:** 10.1186/1471-2164-14-260

**Published:** 2013-04-17

**Authors:** Maria K Kowalczuk, Shreeya Nanda, Elizabeth C Moylan

**Affiliations:** The authors are the Biology Editors for BioMed Central, BioMed Central, 236 Gray’s Inn Road, London, WC1X 8HB United Kingdom

## Abstract

After publication of this article (Fernandez et al., BMC Genomics 2011, **12**:604) it was brought to the Editors’ attention that the data generated by the first author, Ariel Fernandez, seemed anomalous. One of the author’s institutions found that the data were not reproducible from the described methods, but an investigation by the author’s other institution did not find the data or their interpretation suspicious. Given the conflicting conclusions of these investigations, the Editors advise the readers to interpret the data with due caution. We apologize to all affected parties.

## Comment on

Ariel Fernandez, Yun-Huei Tzeng and Sze-Bi Hsu. Subfunctionalization reduces the fitness cost of gene duplication in humans by buffering dosage imbalances. *BMC Genomics* 2011, **12**:604. doi: http://dx.doi.org/10.1186/1471-2164-12-604. URL http://www.biomedcentral.com/1471-2164/12/604.
